# Development and psychometric characteristics of the SCI-QOL Ability to Participate and Satisfaction with Social Roles and Activities item banks and short forms

**DOI:** 10.1179/2045772315Y.0000000028

**Published:** 2015-05

**Authors:** Allen W. Heinemann, Pamela A. Kisala, Elizabeth A. Hahn, David S. Tulsky

**Affiliations:** 1 Center for Rehabilitation Outcomes Research, Rehabilitation Institute of Chicago and Department of Physical Medicine and Rehabilitation, Feinberg School of Medicine, Northwestern University, 345 E. Superior St. Chicago, IL 60611, USA; 2 Center on Assessment Research and Translation, College of Health Sciences and the Department of Physical Therapy, University of Delaware, STAR Health Sciences Complex, 540 South College Avenue, Newark, DE 19713 USA; 3 Department of Medical Social Sciences and Center for Patient-Centered Outcomes, Northwestern University Feinberg School of Medicine, Northwestern University, Chicago, Illinois, USA; 4 Kessler Foundation Research Center, West Orange, New Jersey 07052 USA

**Keywords:** Community participation, Quality of life, Social participation, Spinal cord injuries

## Abstract

**Objective:**

To develop a spinal cord injury (SCI)-focused version of PROMIS and Neuro-QOL social domain item banks; evaluate the psychometric properties of items developed for adults with SCI; and report information to facilitate clinical and research use**.**

**Design:**

We used a mixed-methods design to develop and evaluate Ability to Participate in Social Roles and Activities and Satisfaction with Social Roles and Activities items. Focus groups helped define the constructs; cognitive interviews helped revise items; and confirmatory factor analysis and item response theory methods helped calibrate item banks and evaluate differential item functioning related to demographic and injury characteristics.

**Setting:**

Five SCI Model System sites and one Veterans Administration medical center.

**Participants:**

The calibration sample consisted of 641 individuals; a reliability sample consisted of 245 individuals residing in the community.

**Results:**

A subset of 27 Ability to Participate and 35 Satisfaction items demonstrated good measurement properties and negligible differential item functioning related to demographic and injury characteristics. The SCI-specific measures correlate strongly with the PROMIS and Neuro-QOL versions. Ten item short forms correlate >0.96 with the full banks. Variable-length CATs with a minimum of 4 items, variable-length CATs with a minimum of 8 items, fixed-length CATs of 10 items, and the 10-item short forms demonstrate construct coverage and measurement error that is comparable to the full item bank.

**Conclusion:**

The Ability to Participate and Satisfaction with Social Roles and Activities CATs and short forms demonstrate excellent psychometric properties and are suitable for clinical and research applications.

## Introduction

Participation in social roles and activities, a long term outcome following rehabilitation, is valued highly by persons living with the consequences of spinal cord injury (SCI). Health insurance reform legislation emphasizes the need to measure the long term outcomes of rehabilitation services using standardized measures; such measures are critical to new delivery and payment reform models, including Accountable Care Organizations, bundling demonstrations, the Continuing Care Hospital concept, and the Independence at Home Demonstration program. Needed are long term outcome measures to improve the quality of healthcare and reduce cost without sacrificing care. Thus, this report describes the development of standardized measures of long term rehabilitation outcomes focused on participation in social roles and activities and satisfaction with participation in social roles and activities.

### Participation measurement

The World Health Organization defines participation limitations within the context of the International Classification of Disability and Health (ICF). Participation is defined by many rehabilitation researchers as chapters 6 to 9 of ICF's Activity and Participation domain, those pertaining to Domestic Life, Interpersonal Interactions and Relationships, Major Life Areas, and Community, Social and Civic Life,^1^ and the definition we adopt in this report. Participation measurement is complicated by the World Health Organization's decision to combine activities and participation in a single classification making it impossible to distinguish performance at the individual level (activity) and performance at the societal level (participation). Needed are reliable and valid measures of participation that allow the separation of objective participation performance and subjective satisfaction with participation.

### Research on participation measurement

Magasi and Post^2^ reviewed the content of contemporary participation measures. They identified 8 participation measures developed using classical test theory methods that demonstrated moderate to good validity and reliability, though reporting of measurement information was often incomplete. The measures most often assessed the ICF domains of mobility; domestic life; social interactions; major life domains; and community, social, and civic life.

Dijkers^3^ noted that participation is defined frequently as the primary valued outcome of rehabilitation, but that vague and incomplete definitions and inadequate measurement of participation severely limit clinicians’ and investigators’ ability to measure participation outcomes. He described several major issues related to participation measurement and offers conceptual and methodological guidance.

Whiteneck^1^ observed that the large and growing number of participation measures limits rehabilitation research and practice. He described desirable types and characteristics of participation measures and made recommendations for future participation research. He noted that participation measures vary in regards to specificity, conceptual models, development approach, psychometric properties, dimensionality, objective vs. subjective perspectives, and use of norms He encouraged development of better participation measures built on consensus regarding conceptual models.

Over the last ten years, the National Institutes of Health (NIH) has supported the development of instruments to provide common data elements for clinical research.^4,5^ The Patient Reported Outcomes Measurement Information System (PROMIS)^6^ is a family of instruments that measure patient-reported health status.^4,5^ The National Institutes of Neurological Disorders and Stroke has taken the lead in developing core data sets for neurologic populations, including epilepsy, stroke, and Parkinson's disease among other conditions through the Quality of Life in Neurological Disorders (Neuro-QOL) project.^7^ Neuro-QOL provides a conceptual framework and a common language for clinicians and investigators.

The PROMIS Social Health Workgroup conducted a systematic review of social health definitions, content, and item wording, and implemented a qualitative item review process that included identification of items, development of new items, focus group exploration of domain coverage and cognitive interviews.^8–10^ Validation testing in diverse samples (*n* = 2,208 English; *n* = 644 Spanish) resulted in psychometrically-sound and culturally-appropriate measures of social function, including Ability to Participate in Social Roles and Activities (SRA), and Satisfaction with SRA.^10^ Work also began on social relationships, focusing on social support and social isolation.^8–11^ The extent to which these concepts and measures apply to persons with SCI has not been evaluated.

### Study objectives

The objectives of this study were to (1) develop item banks of social functioning that are relevant to persons with SCI; (2) link the item banks to either the PROMIS or Neuro-QOL (or both) through common items and transform the score to the PROMIS or Neuro-QOL metric; (3) evaluate the psychometric properties of item pools developed for adults with SCI; and (4) report information that facilitates clinical and research use**.**

## Methods

### Item set development

Based on focus group feedback, we identified initially 3 realms that are relevant to social participation: family and friends, leisure, and work. We began with 129 items generated during individual interviews^12,13^ and developed 214 new items based on the focus group transcripts^14^ to form the basis of item banks. Given the work completed by the PROMIS^10^ and Neuro-QOL collaborators, we decided to adopt PROMIS’ domains of Ability to Participate and Satisfaction with SRA. We found these subdomains to be more useful than our initial 3 realms given the assumption of unidimensionality required for item response theory (IRT) analyses and computer adaptive testing (CAT) programming. We binned the items generated from individual interviews and focus groups with 82 Neuro-QOL social domain items into subdomains of Ability to Participate in SRA and Satisfaction with SRA. Two investigators independently assigned items to subdomains; when they disagreed, they discussed and reconciled differences.

Because a goal of the SCI-QOL project was to evaluate the Neuro-QOL's social domain items in an SCI population and supplement them with SCI-relevant items, we prioritized the 38 Neuro-QOL Ability to Participate items and the 44 Satisfaction items. Due to the Neuro-QOL's linkage with PROMIS, this set included 23 PROMIS Satisfaction items. The project team reviewed the remaining items and determined that all but 4 Ability and 4 Satisfaction items were redundant with the Neuro-QOL content. The team winnowed the item pool to 50 Ability and 49 Satisfaction items

Following PROMIS instrument development standards,^15^ we scheduled cognitive debriefing interviews^16^ with individuals with SCI (*n* ≥ 5 per item). Because the Neuro-QOL items had already undergone this extensive review, only the 8 new SCI-QOL social items underwent this high level of review. No items required modification based on cognitive interviews.

Next, we completed a translatability review to ensure that the SCI-QOL items would be amenable to translation into other languages. An experienced team conducted a thorough review of the items to evaluate issues with vocabulary or grammar that could affect the meaning of items translated to Spanish. We modified one Ability to Participate and one Satisfaction item based on translatability feedback. We rephrased ‘I am able to navigate a crowded social situation’ as two new items: ‘I am able to interact with people in social situations’ and ‘I am physically able to move through a crowded room.’ We removed ‘amount of’ from ‘I am satisfied with the amount of control I have over my daily activities.’ Finally, we evaluated the reading level of new items using the Lexile Framework^17^ to assure that they did not exceed a fifth grade level.

### Field testing

We administered the 50 Ability to Participate and 49 Satisfaction items to a sample of individuals with SCI to provide data for IRT calibration. As outlined by Tulsky and colleagues (overview paper, this issue)^13^, 5 SCI Model System sites (Craig Hospital, Kessler Rehabilitation Hospital, Rehabilitation Institute of Chicago, University of Michigan, and University of Washington) and the James J. Peters Veterans Hospital participated in data collection. Interviewers read each question aloud from a computer screen and entered responses into a secure data platform. Institutional Review Boards at each site approved this study. We developed a procedure manual and used it to provide interviewers with training to certify their competence.

### Calibration sample

A total of 641 individuals with traumatic SCI completed the two item sets. Inclusion criteria were SCI due to traumatic event, age 18 or older at the time of study participation, and ability to read and understand English. There were no further exclusion criteria. Level and severity of injury were confirmed through medical record review.

### Reliability sample

An independent sample of 245 individuals from 4 SCI Model Systems centers (University of Michigan, Kessler Institute for Rehabilitation, Rehabilitation Institute of Chicago, Craig Hospital) completed the item banks twice as part of a larger project in which we tested SCI-QOL CATs and short forms at multiple intervals.^12^ Each site's Institutional Review Board reviewed and approved the study protocol. Eligibility criteria were similar to the calibration study: traumatic SCI, 18 years or older, and ability to read, speak, and understand English fluently. We stratified the sample by level and completeness of injury as well as time since injury (≤2 years, 2>years). Participants were community-dwelling and sustained SCI more than 4 months before the assessment. Our goal was to have participants complete the second assessment 7–14 days after the first assessment.

### Data analysis

We evaluated dimensionality of the Ability to Participate and Satisfaction item sets using confirmatory factor analysis with MPLUS version 6. Criteria for unidimensionality were a comparative fit index (CFI) and a Tucker-Lewis Index (TLI) >0.9, a root mean square error of approximation (RMSEA) <0.08 for good fit and <0.05 for excellent fit.^18^

### 2-parameter item response theory analyses

We used a graded response IRT model^19^ using MULTILOG software and examined the S-X^2^ model fit statistics using the IRTFIT macro program.^20^ We removed items that demonstrated local item dependence (residual correlation >|0.2|), significant (P < 0.05), misfit (S-X^2^ test),^21^ or differential item functioning (DIF)^22^ due to sex, age (<50 vs. ≥50), education (some college or less vs. college degree or higher), level of injury (paraplegia vs. tetraplegia), injury severity (complete vs. incomplete), and time post injury (<1 year vs.≥1 year).

We transformed SCI-QOL item parameters to Neuro-QOL's general population metric following the procedure reported by Tulsky and colleagues.^12^

## Results

### Calibration sample characteristics

Tulsky and colleagues describe the demographic characteristics of the focus group participants.^13^ Table 1 shows that the sample of 641 respondents was middle-aged (mean 42.9 years old) with a range from 18 to 91 years. Reflecting the epidemiology of SCI, men outnumbered women 3 to 1; the largest group was single and never married. The sample reflects the US population in terms of ethnicity; racial minority groups were somewhat over-represented. Educational attainment included high school or less education (37.6%), some college (33.9%), and a baccalaureate degree or higher (28.6%). Household income ranged from less than $20,000 (26.4%), 20,000 to $74,999 (36.4%), to $75,000 or more (22.5%); 14.8% did not know their household income or declined to report it. Most participants (89.6%) resided in a private residence. The average age at SCI was 35.6 years (SD = 15.6). Primary means of mobility included walking (33.2%), propelling a manual wheelchair (53.4%), and using a power wheelchair (40.4%).

**Table 1:** Demographic Characteristics of the Calibration Sample

Variable	Social Participation Domain Sample *N* = 641 Mean (SD), N (%)
Age (years)	42.9 (15.3)
Age at injury (years)	35.9 (16.9)
Sex	
Male	496 (77.4%)
Female	145 (22.6%)
Ethnicity	
Hispanic	66 (10.3%)
Non-Hispanic	571 (89.1%)
Not reported	4 (0.7%)
Race	
Caucasian	460 (72.6%)
African-American	110 (17.4%)
Asian	6 (0.9%)
American Indian/Alaska Native or Native Hawaiian/Pacific Islander	5 (0.8%)
More than one race	7 (1.1%)
Other	42 (6.6%)
Not reported	11 (1.8%)
Time Since Injury	7.1 (9.8)
<1 year post injury	139 (21.7%)
1–3 years post injury	192 (30.0%)
>3 years post injury	310 (48.4%)
Injury Level and Completeness	
Paraplegia Complete	163 (25.4%)
Paraplegia Incomplete	120 (18.7%)
Tetraplegia Complete	132 (20.6%)
Tetraplegia Incomplete	222 (34.6%)

### Item characteristics

Table 2 shows descriptive statistics for the retained Ability to Participate items Table 3 shows the same information for the Satisfaction items

**Table 2:** Item Statistics for Retained Ability to Participate in Social Roles and Activities Items

Item ID	Item Stem	Mean	SD	% at Min	% at Max
**NQPRF01**	**I can keep up with my family responsibilities.**	**3.80**	**1.176**	**6.2**	**34.8**
NQPRF02	I have trouble meeting the needs of my family.	3.88	1.176	5.0	40.4
NQPRF03	I am able to do all of my regular family activities.	3.70	1.170	5.8	31.4
NQPRF04	I have to limit my regular family activities.	3.75	1.213	6.2	36.0
NQPRF05	I am able to do all of the family activities that people expect me to do.	3.75	1.163	5.2	33.6
**NQPRF06**	**I am able to do all of the family activities that I want to do.**	**3.52**	**1.239**	**8.0**	**28.0**
**NQPRF08**	**I am able to socialize with my friends.**	**4.06**	**1.105**	**3.6**	**47.0**
NQPRF09	I am able to do all of my regular activities with friends.	3.45	1.247	8.4	25.9
**NQPRF11**	**I can do everything for my friends that I want to do.**	**3.26**	**1.280**	**10.0**	**22.9**
NQPRF12	I am able to do all of the activities with friends that people expect me to do.	3.56	1.192	6.1	28.2
**NQPRF14**	**I am able to do all of the activities with friends that I want to do.**	**3.35**	**1.263**	**9.2**	**24.0**
NQPRF16	I have to limit the things I do for fun at home (like reading; listening to music; etc.).	3.63	1.306	8.0	36.0
**NQPRF17**	**I can keep up with my social commitments.**	**3.83**	**1.068**	**3.4**	**31.5**
**NQPRF18**	**I am able to do all of my regular leisure activities.**	**3.69**	**1.186**	**5.8**	**32.1**
NQPRF19	I have to limit my hobbies or leisure activities.	3.30	1.200	8.7	20.6
NQPRF20	I am able to do my hobbies or leisure activities.	3.63	1.153	5.6	28.1
NQPRF21	I am able to do all of the community activities that I want to do.	3.16	1.351	15.5	20.7
NQPRF22	I am able to do all of the leisure activities that people expect me to do.	3.56	1.230	7.5	28.6
NQPRF23	I have to do my hobbies or leisure activities for shorter periods of time than usual for me.	3.44	1.266	8.0	28.7
NQPRF26	I am able to participate in leisure activities.	3.64	1.136	4.7	28.1
**NQPRF27**	**I can do all the leisure activities that I want to do.**	**3.33**	**1.292**	**10.5**	**24.2**
NQPRF29	I am able to go out for entertainment as much as I want.	3.29	1.327	11.7	24.7
NQPRF30	I have to limit the things I do for fun outside my home.	3.07	1.302	15.1	18.0
NQPRF31	I am doing fewer social activities with groups of people than usual for me.	3.34	1.321	11.8	25.3
**NQPRF32**	**I am able to perform my daily routines.**	**4.02**	**1.140**	**5.6**	**44.9**
**NQPRF34**	**I can keep up with my work responsibilities.**	**3.60**	**1.289**	**10.8**	**30.5**
NQPRF40	I have trouble doing my regular chores or tasks.	3.46	1.278	9.8	28.3

*Context for all items was ‘In the past 7 days….’ Response set was: Never/Rarely/Sometimes/Often/Always.

Positively worded items were scored 1–5 and negatively worded items were scored 5–1.

**Bold text** indicates items selected for the short form.

Note: For this item bank, all items are from Neuro-QOL.

**Table 3:** Item Statistics for Retained Satisfaction with Social Roles and Activities Items

Item ID	Item Stem	Mean	SD	% at Min	% at Max
NQSAT01	I feel that my family is disappointed in my ability to socialize with them.	4.22	1.107	3.6	58.3
**NQSAT02**	**I am disappointed in my ability to meet the needs of my family.**	**3.72**	**1.275**	**7.6**	**37.9**
**NQSAT03**	**I am bothered by my limitations in regular family activities.**	**3.59**	**1.279**	**9.1**	**31.3**
NQSAT08	I am satisfied with my current level of activity with family members.	3.53	1.221	8.3	25.7
NQSAT10	I feel that my friends are disappointed in my ability to socialize with them.	4.13	1.112	3.1	53.1
NQSAT11	I am disappointed in my ability to meet the needs of my friends.	3.80	1.160	4.8	35.9
NQSAT12	I am disappointed in my ability to do things for my friends.	3.59	1.220	8.1	27.7
**NQSAT13**	**I am disappointed in my ability to socialize with friends.**	**3.81**	**1.300**	**7.5**	**44.1**
NQSAT14	I am bothered by limitations in my regular activities with friends.	3.40	1.290	10.2	25.9
NQSAT15	I am disappointed in my ability to keep in touch with others.	3.87	1.169	4.7	40.5
NQSAT22	I feel that others are disappointed in my ability to do community activities.	4.26	1.043	2.3	59.0
NQSAT23	I am disappointed in my ability to socialize with my family.	3.97	1.223	5.9	48.5
NQSAT24	I am disappointed in my ability to do leisure activities.	3.49	1.286	9.7	28.1
NQSAT25	I am bothered by limitations in doing my hobbies or leisure activities.	3.22	1.362	14.8	23.6
NQSAT36	I am disappointed in my ability to perform my daily routines.	3.76	1.218	6.1	37.2
NQSAT37	I am disappointed in my ability to work (include work at home).	3.45	1.457	15.8	34.9
NQSAT38	I am bothered by limitations in performing my daily routines.	3.40	1.280	10.5	25.3
**NQSAT39**	**I am disappointed in my ability to take care of personal and household responsibilities.**	**3.60**	**1.326**	**10.4**	**34.8**
**NQSAT40**	**I am bothered by limitations in performing my work (include work at home).**	**3.39**	**1.341**	**12.8**	**27.3**
NQSAT46	I am satisfied with my ability to do household chores or tasks.	3.15	1.359	16.6	21.2
RSATIS_56	I am satisfied with the amount of physical contact I have with others.	3.30	1.265	10.3	21.8
SRPSAT05	I am satisfied with the amount of time I spend doing leisure activities.	3.47	1.198	8.6	23.0
SRPSAT06	I am satisfied with my ability to do things for my family.	3.31	1.252	10.8	20.0
SRPSAT08	I feel good about my ability to do things for my family.	3.52	1.225	8.1	26.1
**SRPSAT10**	**I am satisfied with my current level of social activity.**	**3.36**	**1.253**	**10.0**	**21.5**
SRPSAT20	I am satisfied with my ability to do things for my friends.	3.20	1.248	11.7	18.3
**SRPSAT23**	**I am satisfied with my ability to do leisure activities.**	**3.34**	**1.193**	**8.4**	**19.3**
**SRPSAT25**	**I am satisfied with my current level of activities with my friends.**	**3.29**	**1.238**	**10.0**	**19.5**
SRPSAT33	I am satisfied with my ability to do things for fun outside my home.	3.31	1.294	11.4	23.1
SRPSAT36	I am happy with how much I do for my friends.	3.28	1.228	9.7	19.5
SRPSAT38	I am satisfied with the amount of time I spend performing my daily routines.	3.44	1.256	9.4	25.4
**SRPSAT48**	**I am satisfied with my ability to do things for fun at home (like reading; listening to music; etc.).**	**3.77**	**1.130**	**4.5**	**32.7**
**SRPSAT49**	**I am satisfied with my ability to perform my daily routines.**	**3.52**	**1.196**	**7.6**	**24.0**
SRPSAT50	I am satisfied with my ability to meet the needs of those who depend on me.	3.39	1.240	9.8	22.2
SRPSAT52	I am satisfied with my ability to do all of the leisure activities that are really important to me.	3.37	1.273	10.5	23.1

*Context for all items was ‘In the past 7 days….’ Response set was: Never/ Rarely/ Sometimes/ Often/ Always.

Positively worded items were scored 1–5 and negatively worded items were scored 5–1.

**Bold text** indicates items selected for the short form.

Note: For this item bank, all ‘NQ’ items are from Neuro-QOL, all ‘SRP’ items are PROMIS items embedded in Neuro-QOL, and items beginning with ‘RSATIS’ were newly written as a part of SCI-QOL.

### Confirmatory factory analysis

#### Ability to participate

We deleted 23 items due to a significant χ^2^ value indicating misfit (9), bimodal distributions (3), residual correlations greater than |0.2| indicating local item dependence (11), or *r*^2^ values less than 0.3 indicating low item-total correlation (1). Some of the Neuro-QOL items pertaining to work demonstrated a bimodal distribution of responses, suggesting that people who are employed complete the items in one manner and people who are unemployed complete the items differently. The items displayed misfit, local dependence, and differential item functioning (DIF), all of which could bias the results and performance of the scale in this population. Therefore, we removed several employment related items

*Satisfaction*: We deleted 14 items due to bimodal distributions (6), local item dependence (11), and misfit (2).

### IRT calibrations

Table 4 shows the calibration statistics, including slopes and response category thresholds, for the 27 retained Ability to Participate items; all of them are Neuro-QOL items. These calibration parameters have been optimized for an SCI population but, as described below, have been transformed to the Neuro-QOL metric so they are interpretable as Neuro-QOL scores.

**Table 4:** Item Calibration Statistics for Ability to Participate in Social Roles and Activities

		Item Response Theory Calibration Statistics				
Item ID	Item Stem	Slope	Threshold 1	Threshold 2	Threshold 3	Threshold 4
**NQPRF01**	**I can keep up with my family responsibilities.**	**3.38698**	**−1.69617**	**−1.33837**	**−0.80578**	**−0.20366**
NQPRF02	I have trouble meeting the needs of my family.	2.28641	−2.08061	−1.52406	−0.89687	−0.27276
NQPRF03	I am able to do all of my regular family activities.	4.45561	−1.58675	−1.23058	−0.66923	−0.15992
NQPRF04	I have to limit my regular family activities.	3.62011	−1.63942	−1.24675	−0.69186	−0.22756
NQPRF05	I am able to do all of the family activities that people expect me to do.	3.61330	−1.72550	−1.30100	−0.70532	−0.18349
**NQPRF06**	**I am able to do all of the family activities that I want to do.**	**4.87786**	**−1.45347**	**−1.08202**	**−0.55159**	**−0.10731**
**NQPRF08**	**I am able to socialize with my friends.**	**3.25055**	**−1.90988**	**−1.50039**	**−0.94773**	**−0.43299**
NQPRF09	I am able to do all of my regular activities with friends.	4.45221	−1.46098	−1.03978	−0.50505	−0.04327
**NQPRF11**	**I can do everything for my friends that I want to do.**	**4.24692**	**−1.41618**	**−0.90393**	**−0.37758**	**0.03742**
NQPRF12	I am able to do all of the activities with friends that people expect me to do.	5.39507	−1.52070	−1.08910	−0.53523	−0.10539
**NQPRF14**	**I am able to do all of the activities with friends that I want to do.**	**5.65722**	**−1.37058**	**−0.93203**	**−0.43875**	**−0.02455**
NQPRF16	I have to limit the things I do for fun at home (like reading, listening to music, etc.).	2.55206	−1.75140	−1.19259	−0.63541	−0.17539
**NQPRF17**	**I can keep up with my social commitments.**	**3.68270**	**−1.84171**	**−1.36553**	**−0.78896**	**−0.13298**
**NQPRF18**	**I am able to do all of my regular leisure activities.**	**5.15416**	**−1.53659**	**−1.15718**	**−0.63584**	**−0.18498**
NQPRF19	I have to limit my hobbies or leisure activities.	3.22206	−1.56832	−1.07768	−0.35458	0.14979
NQPRF20	I am able to do my hobbies or leisure activities.	5.30848	−1.52805	−1.16511	−0.61048	−0.10432
NQPRF21	I am able to do all of the community activities that I want to do.	3.27447	−1.30228	−0.86801	−0.38784	0.15000
NQPRF22	I am able to do all of the leisure activities that people expect me to do.	3.65736	−1.57175	−1.13383	−0.58851	−0.06034
NQPRF23	I have to do my hobbies or leisure activities for shorter periods of time than usual for me.	2.65949	−1.71476	−1.13854	−0.44568	−0.01487
NQPRF26	I am able to participate in leisure activities.	4.20314	−1.69697	−1.23127	−0.60722	−0.07859
**NQPRF27**	**I can do all the leisure activities that I want to do.**	**5.01492**	**−1.34415**	**−0.91530**	**−0.44223**	**−0.01657**
NQPRF29	I am able to go out for entertainment as much as I want.	3.31953	−1.42235	−0.92968	−0.43537	0.03210
NQPRF30	I have to limit the things I do for fun outside my home.	3.04342	−1.34217	−0.86476	−0.25417	0.24905
NQPRF31	I am doing fewer social activities with groups of people than usual for me.	2.10814	−1.69043	−1.11999	−0.45972	0.14476
**NQPRF32**	**I am able to perform my daily routines.**	**3.54175**	**−1.68835**	**−1.44811**	**−0.96290**	**−0.40468**
**NQPRF34**	**I can keep up with my work responsibilities.**	**3.32780**	**−1.47119**	**−1.21180**	**−0.70031**	**−0.11689**
NQPRF40	I have trouble doing my regular chores or tasks.	3.14497	−1.53005	−1.12830	−0.49027	−0.03716

*Context for all items was ‘In the past 7 days….’ Response set was Never/Rarely/Sometimes/Often/Always.

Positively worded items were scored 1–5 and negatively worded items were scored 5–1.

**Bold Font** indicates the items selected for the short form.

Note: For this item bank, all items are from Neuro-QOL.

Table 5 shows the calibration statistics, including slopes and response category thresholds, for the 35 retained Satisfaction items; 34 of them are Neuro-QOL items and 15 of these are PROMIS items. Similar to the Ability to Participate domain, these calibration parameters are optimized for an SCI population and transformed to the Neuro-QOL metric.

**Table 5:** Item Calibration Statistics for Satisfaction with Social Roles and Activities items

		Item Response Theory Calibration Statistics				
Item ID	Item Stem	Slope	Threshold 1	Threshold 2	Threshold 3	Threshold 4
NQSAT01	I feel that my family is disappointed in my ability to socialize with them.	3.35305	−1.76646	−1.42376	−0.95896	−0.62579
**NQSAT02**	**I am disappointed in my ability to meet the needs of my family.**	**4.67405**	**−1.35290**	**−1.02772**	**−0.64174**	**−0.31288**
**NQSAT03**	**I am bothered by my limitations in regular family activities.**	**5.18066**	**−1.28224**	**−0.97999**	**−0.60857**	**−0.23464**
NQSAT08	I am satisfied with my current level of activity with family members.	3.89422	−1.40092	−1.03959	−0.57823	−0.06090
NQSAT10	I feel that my friends are disappointed in my ability to socialize with them.	2.66505	−2.03708	−1.53325	−0.94353	−0.54065
NQSAT11	I am disappointed in my ability to meet the needs of my friends.	4.58130	−1.50867	−1.12527	−0.68499	−0.27646
NQSAT12	I am disappointed in my ability to do things for my friends.	4.40691	−1.34056	−1.01411	−0.59774	−0.12814
**NQSAT13**	**I am disappointed in my ability to socialize with friends.**	**4.85035**	**−1.34938**	**−1.03511**	**−0.66497**	**−0.40810**
NQSAT14	I am bothered by limitations in my regular activities with friends.	5.32247	−1.21043	−0.86141	−0.48200	−0.12119
NQSAT15	I am disappointed in my ability to keep in touch with others.	3.88820	−1.58517	−1.20213	−0.71368	−0.33309
NQSAT22	I feel that others are disappointed in my ability to do community activities.	2.85180	−2.05523	−1.56600	−1.02878	−0.65221
NQSAT23	I am disappointed in my ability to socialize with my family.	4.31327	−1.46979	−1.18263	−0.77768	−0.47741
NQSAT24	I am disappointed in my ability to do leisure activities.	5.34836	−1.22840	−0.90100	−0.54456	−0.16428
NQSAT25	I am bothered by limitations in doing my hobbies or leisure activities.	4.31114	−1.12836	−0.80040	−0.42280	−0.04591
NQSAT36	I am disappointed in my ability to perform my daily routines.	5.76091	−1.35427	−1.02765	−0.64515	−0.31782
NQSAT37	I am disappointed in my ability to work (include work at home).	3.83935	−1.13296	−0.88505	−0.55172	−0.24865
NQSAT38	I am bothered by limitations in performing my daily routines.	4.12626	−1.27741	−0.93075	−0.48001	−0.07122
**NQSAT39**	**I am disappointed in my ability to take care of personal and household responsibilities.**	**4.61330**	**−1.23989**	**−0.97548**	**−0.57383**	**−0.26200**
**NQSAT40**	**I am bothered by limitations in performing my work (include work at home).**	**4.26219**	**−1.18227**	**−0.90062**	**−0.49518**	**−0.11926**
NQSAT46	I am satisfied with my ability to do household chores or tasks.	3.90832	−1.11308	−0.81290	−0.38443	0.02383
RSATIS_56	I am satisfied with the amount of physical contact I have with others.	4.14670	−1.27285	−0.87184	−0.42199	0.01768
SRPSAT05	I am satisfied with the amount of time I spend doing leisure activities.	5.23141	−1.27206	−0.98520	−0.51028	−0.05344
SRPSAT06	I am satisfied with my ability to do things for my family.	3.60012	−1.33439	−0.93486	−0.48032	0.07441
SRPSAT08	I feel good about my ability to do things for my family.	5.14478	−1.29539	−0.96559	−0.55667	−0.11105
**SRPSAT10**	**I am satisfied with my current level of social activity.**	**5.40839**	**−1.21257**	**−0.86219**	**−0.48996**	**−0.03007**
SRPSAT20	I am satisfied with my ability to do things for my friends.	5.01028	−1.18557	−0.84451	−0.39558	0.04782
**SRPSAT23**	**I am satisfied with my ability to do leisure activities.**	**5.36713**	**−1.26659**	**−0.89977**	**−0.48151**	**0.01303**
**SRPSAT25**	**I am satisfied with my current level of activities with my friends.**	**5.35026**	**−1.21983**	**−0.83883**	**−0.44301**	**0.01219**
SRPSAT33	I am satisfied with my ability to do things for fun outside my home.	5.03527	−1.18474	−0.85658	−0.46312	−0.06514
SRPSAT36	I am happy with how much I do for my friends.	5.28855	−1.22935	−0.85640	−0.41995	0.02740
SRPSAT38	I am satisfied with the amount of time I spend performing my daily routines.	2.63182	−1.55189	−1.09797	−0.50312	0.04275
**SRPSAT48**	**I am satisfied with my ability to do things for fun at home (like reading, listening to music, etc.).**	**4.30906**	**−1.54659**	**−1.16270**	**−0.67206**	**−0.20534**
**SRPSAT49**	**I am satisfied with my ability to perform my daily routines.**	**5.83776**	**−1.27579**	**−0.95649**	**−0.56670**	**−0.09144**
SRPSAT50	I am satisfied with my ability to meet the needs of those who depend on me.	4.99866	−1.24185	−0.92277	−0.51506	−0.03948
SRPSAT52	I am satisfied with my ability to do all of the leisure activities that are really important to me.	4.97834	−1.21830	−0.87383	−0.49545	−0.06032

*Context for all items was ‘In the past 7 days….’ Response options were Not at all/A little bit/Somewhat/Quite a bit/Very much.

Positively worded items were scored 1–5 and negatively worded items were scored 5–1.

**Bold font** indicates the items selected for the short form.

Note: For this item bank, all ‘NQ’ items are from Neuro-QOL, all ‘SRP’ items are PROMIS items embedded in Neuro-QOL, and items beginning with ‘RSATIS’ were newly written as a part of SCI–QOL.

### Differential item functioning

Seven Ability to Participate items demonstrated statistically significant DIF as did 8 Satisfaction items. When we examined the DIF effect sizes, the practical effects were negligible; thus, we decided to retain them.

### Transformation to Neuro-QOL metric

We computed SCI-specific calibrations using the SCI-QOL calibration sample, then we linked these scores to the Neuro-QOL calibrations that had been developed using a general population reference group using the items that are common to SCI-QOL and Neuro-QOL. We utilized Stocking-Lord equating methods^23^ to calculate transformation slope and intercept parameters. We applied these parameters to create linear transformations so that the SCI-QOL measure maps to the Neuro-QOL metric and scores are reported in terms of general population norms and are equivalent to Neuro-QOL scores. Table 6 shows how the mean scores for each were transformed. SCI-QOL scores are about 5 T-score points lower than Neuro-QOL scores and have less variance

**Table 6:** Scoring Before and After Transformation

Bank	N	T-score Before Transformation (Calibration Parameters)		T-score After Transformation (Transformed Parameters)	
		Mean	S.D.	Mean	S.D.
Ability to Participate	641	50.46	9.76	45.42	6.57*
Satisfaction with SRA	641	50.72	9.77	45.44	5.59*

*Decreased standard deviation may be a result of linking to the general population and to the nature of the measures.

### Test information function and reliability

**Figure 1:**
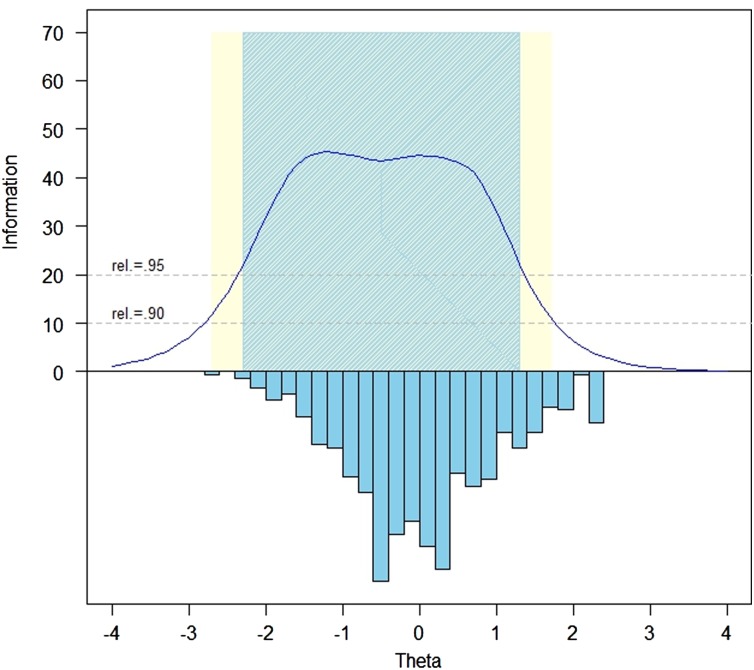
Ability to Participate Item Bank Information and Precision (*i* = 27).

**Figure 2:**
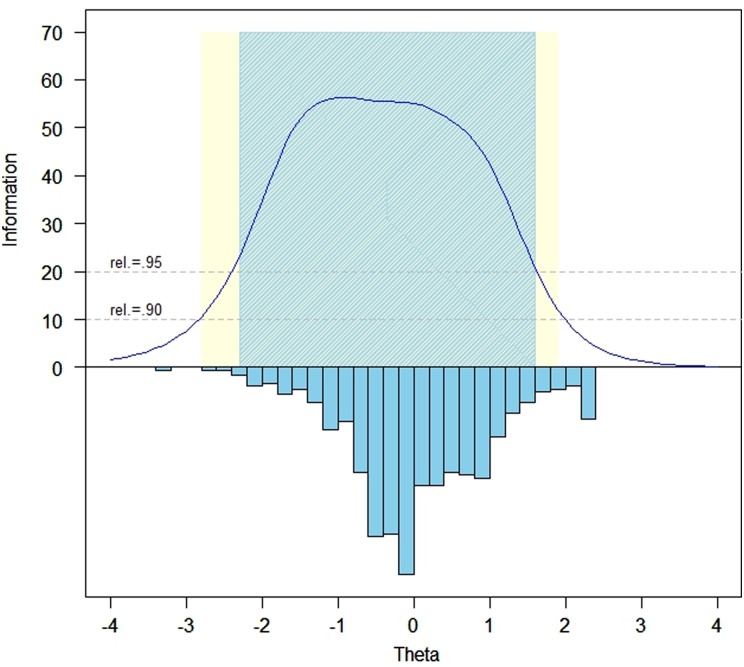
Satisfaction with SRA–Item bank information and precision (*i* = 35).

Fig. 1 illustrates the test information function across the range of the Ability to Participate measure; Fig. 2 illustrates the same function for the Satisfaction items. Reliability exceeds 0.95 within a range of –2.3 to +1.2 theta for Ability to Participate and –2.3 and +1.6 theta for Satisfaction.

### Short form item selection

We selected items to comprise a fixed-length short form version of each item bank for situations in which CAT administration is not practical. First, we reviewed item locations and discrimination. Then, we assigned items into quintiles based on location and selected 1 to 2 items within each quintile with the highest slope. We considered clinical relevance, wording, and similarity to other candidate items. Our goal was to maximize the diversity of short form item content. Following PROMIS naming conventions, the short forms are titled Ability to Participate in SRA SF10a and Satisfaction with SRA SF10a, respectively.

**Figure 3:**
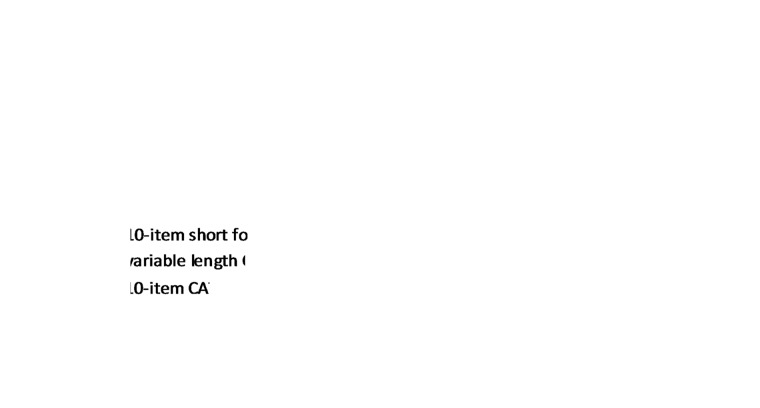
Reliability of Ability to Participate full bank, 10-item short form, variable-length CAT, and 10-item fixed-length CAT.

**Figure 4:**
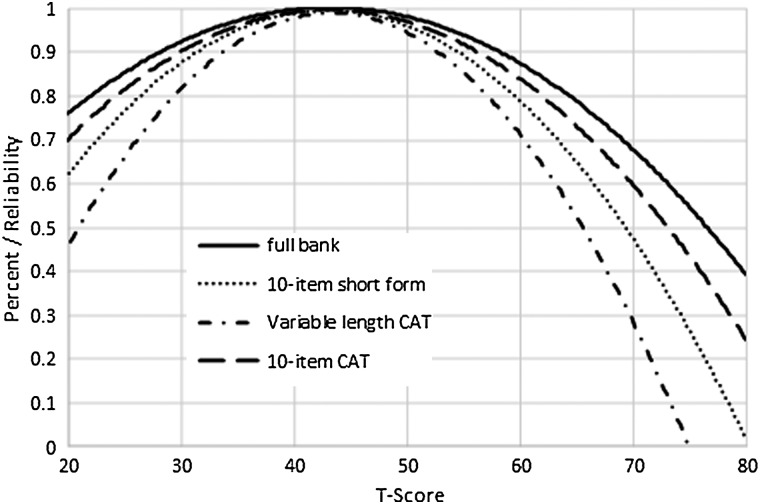
Reliability of Satisfaction with SRA full bank, 10-item short form, variable-length CAT, and 10-item fixed-length CAT.

Figure 3 illustrates the reliability of the Ability to Participate SF10a vs. a variable length CAT (set to the default minimum of 4 items, maximum of 12 items, and maximum standard error 0.3), a 10-item fixed-length CAT, and the full item bank. Reliability exceeds 0.80 between T-scores of 20 and 60. Figure 4 illustrates reliability values for the various modes of administration of the Satisfaction bank. Reliability exceeds 0.80 between T-scores of 26 and 60.

Table 7 shows the breadth of coverage for the Ability to Participate SF10a compared with 4- and 8-item minimum variable-length CATs, a 10-item fixed length CAT, and the full item bank. The correlation between the 10-item Ability to Participate SF10a with the full item bank exceeds 0.96. Table 8 shows the same information for the Satisfaction SF10a. T-score means were essentially identical as was the range of scores and standard errors. The SF10a has somewhat greater ceiling effects than the other versions.

**Table 7:** Ability to Participate: Accuracy of Variable- and Fixed-Length CAT and 10-Item Short Form

Mode	N	No. Items Administered			Max	%Min	%Max	Correlation with Full Bank
		Mean	SD	Min				
Variable-Length CAT (min 4)	641	4.82	2.23	4	12	84.40	7.49	0.954
Variable-Length CAT (min 8)	641	8.34	1.07	8	12	90.95	7.49	0.977
10-Item Fixed-Length CAT	641	10	0	10	10	n/a	n/a	0.979
10-Item Short Form	641	10	0	10	10	n/a	n/a	0.963

n/a: not applicable.

**Table 8:** Satisfaction with SRA: Accuracy of Variable- and Fixed-Length CAT and 10-item Short Form

Mode	N	No. Items Administered			Max	%Min	%Max	Correlation with Full Bank
		Mean	SD	Min				
Variable-Length CAT (min 4)	641	4.53	1.81	4	12	1.87	4.68	0.940
Variable-Length CAT (min 8)	641	8.20	0.86	8	12	94.70	4.68	0.966
10-Item Fixed-Length CAT	641	10	0	10	10	n/a	n/a	0.967
10-Item Short Form	641	10	0	10	10	n/a	n/a	0.961

n/a: not applicable.

Table 9 provides means, ranges, and standard errors for the Ability to Participate variable length CATs, 10-item CAT, 10-item short form, and full bank. Means and standard deviations, ranges, percent of the sample at the ceiling and floor, and standard errors are essentially identical.

**Table 9:** Ability to Participate: Breadth of Content Coverage for Variable Length CAT, Fixed Length CAT, 10-item Short Form, and Full Item Bank

Mode	N	T Score				Standard Error	
		Mean ± SD	Range	% Ceiling	% Floor	Mean ± SD	Range
Variable-Length CAT (min 4)	641	45.63 ± 6.69	27.80–63.71	3.12	0.16	0.209 ± 0.08	0.156–0.550
Variable-Length CAT (min 8)	641	45.49 ± 6.67	27.80–63.71	3.12	0.16	0.166 ± 0.09	0.115–0.549
10-Item Fixed-Length CAT	641	45.49 ± 6.71	28.29–63.40	3.43	0.16	0.157 ± 0.09	0.106–0.554
10-Item Short Form	641	45.66 ± 6.87	28.30–61.10	6.85	0.16	0.188 ± 0.19	0.120–0.570
Full Bank	641	45.42 ± 6.57	27.10–64.50	2.50	0.16	0.188 ± 0.18	0.120–0.570

Table 10 provides the same information for Satisfaction with SRA. Again, means and standard deviations, ranges, percent of the sample at the ceiling and floor, and standard errors are essentially identical across different versions.

**Table 10:** Satisfaction with SRA: Breadth of Content Coverage for Variable Length CATs, Fixed Length CAT, 10-Item Short Form, and Full Item Bank

Mode	N	T Score				Standard Error	
		Mean ± SD	Range	% Ceiling	% Floor	Mean ± SD	Range
Variable-Length CAT (min 4)	641	45.84 ± 5.80	24.17–62.29	3.43	0.16	0.198 ± 0.078	0.146–0.544
Variable-Length CAT (min 8)	641	45.64 ± 5.79	24.17–62.29	3.43	0.16	0.151 ± 0.086	0.104–0.544
10-Item Fixed-Length CAT	641	45.66 ± 5.86	24.37–62.00	3.90	0.16	0.141 ± 0.093	0.093–0.550
10-Item Short Form	641	45.62 ± 5.81	28.3–60.5	5.15	0.31	0.159 ± 0.112	0.100–0.570
Full Bank	641	45.44 ± 5.59	23.62–63.21	2.34	0.16	0.091 ± 0.082	0.059–0.529

Tables 11 and 12 provide the raw to scaled score conversions for the 10-item short forms for Ability to Participate and Satisfaction with SRA, respectively.

**Table 11:** Lookup Table for SCI-QOL v1.0 Ability to Participate in Social Roles and Activities SF10a

Raw score	Scaled (T) score	Standard error
10	25.1	4.0
11	28.7	2.7
12	30.0	2.5
13	31.2	2.2
14	32.1	2.0
15	33.0	1.9
16	33.7	1.8
17	34.4	1.7
18	35.0	1.6
19	35.6	1.6
20	36.1	1.5
21	36.7	1.5
22	37.2	1.5
23	37.7	1.5
24	38.2	1.5
25	38.7	1.5
26	39.2	1.5
27	39.7	1.5
28	40.2	1.5
29	40.7	1.5
30	41.2	1.5
31	41.7	1.5
32	42.2	1.5
33	42.7	1.5
34	43.2	1.5
35	43.7	1.5
36	44.3	1.5
37	44.8	1.5
38	45.3	1.5
39	45.9	1.5
40	46.5	1.5
41	47.1	1.5
42	47.7	1.5
43	48.3	1.5
44	49.0	1.6
45	49.7	1.7
46	50.6	1.9
47	51.6	2.0
48	53.0	2.4
49	54.9	2.9
50	61.1	5.6

**Table 12:** Lookup Table for SCI-QOL v1.0 Satisfaction with Social Roles and Activities SF10a

Raw score	Scaled (T) score	Standard error
10	28.3	4.1
11	32.4	2.2
12	33.6	2.0
13	34.6	1.7
14	35.4	1.6
15	36.1	1.4
16	36.7	1.4
17	37.2	1.3
18	37.7	1.3
19	38.1	1.2
20	38.6	1.2
21	39.0	1.2
22	39.4	1.2
23	39.8	1.2
24	40.2	1.2
25	40.6	1.2
26	41.0	1.2
27	41.4	1.2
28	41.8	1.2
29	42.2	1.2
30	42.5	1.2
31	42.9	1.2
32	43.3	1.2
33	43.7	1.2
34	44.1	1.2
35	44.5	1.2
36	44.9	1.2
37	45.3	1.2
38	45.8	1.2
39	46.2	1.2
40	46.7	1.3
41	47.2	1.3
42	47.7	1.3
43	48.2	1.3
44	48.8	1.4
45	49.4	1.5
46	50.2	1.6
47	51.1	1.8
48	52.2	2.1
49	53.8	2.4
50	60.5	5.7

### Test-retest reliability

The 245 retest participants completed the second testing 7–14 days following the first assessment. The Pearson's test-retest correlation for Ability to Participate was 0.75 and 0.78 for Satisfaction (P < 0.001), accounting for more than 50% shared variance. The ICC (2,1) for Ability to Participate was 0.74; 95%CI = 0.67, 0.79 and for Satisfaction the ICC (2,1) was 0.77; 95% CI = 0.72, 0.82.

## Discussion

The objectives of this study were to (1) develop item banks measuring social domains of functioning in an SCI population; (2) link the measure(s) to PROMIS and Neuro-QOL; (3) evaluate the psychometric properties of item pool developed for adults with SCI; and (4) report information that facilitates clinical and research use. The 27-item Ability to Participate and a 35-item Satisfaction with Participation item bank fulfill these objectives.

The SCI-QOL Ability to Participate and Satisfaction with SRA banks are optimized versions of the Neuro-QOL/PROMIS v1.0 social item banks for use by individuals with SCI. Items calibrations were developed using a large, heterogeneous, and representative sample. When administered as a CAT, items will be selected based on their functioning in an SCI sample. Similarly, item content that is tailored to an SCI population could be added and items that do not function well in an SCI population could be identified and removed. For example, several Neuro-QOL/PROMIS items related to employment had bimodal distributions or demonstrated poor model fit, local dependence, or differential item functioning, likely due to high rates of un- and under-employment in this sample. Given the importance of employment issues, the research team was presented with a dilemma of retaining content that could bias the overall measurement scale or remove poorly functioning items We decided to remove most problematic items at the expense of reducing content coverage. We recommend that future research develop item banks focused exclusively on employment issues. We excluded all but one (RSATIS_56, ‘I am satisfied with the amount of physical contact I have with others’) of the newly developed items from the final item banks. With the exception of the employment items as described above, the PROMIS and Neuro-QOL Ability to Participate and Satisfaction with SRA items describe most of the participation-related issues that are relevant for individuals with SCI.

Another significant advancement of this project is the development of calibrations optimized for individuals with SCI and items that are relevant and appropriate for this population. No other participation measure is customized to persons with SCI and used IRT methods in their development. Transformation to the Neuro-QOL metric enables direct comparison of SCI-QOL with Neuro-QOL social bank scores from other disability samples. Notably, the Ability to Participate and Satisfaction with SRA banks demonstrate lower mean scores (∼45 vs. ∼50) and restricted range (SD ∼6 vs. SD ∼10) when transformed to reflect the general population norms. This result suggests that people with SCI are limited in both their ability to participate and satisfaction with participation, and that the range of engagement in social roles and activities is more limited in individuals in SCI than in the general population. Social participation is an important issue for clinicians to target for clinical services, and for investigators to develop effective interventions.

The psychometric properties of the SCI-QOL Ability to Participate and Satisfaction CATs and short forms are excellent. Full item banks and short forms are available as PDF files from the authors; CATs may be administered through the NIH Assessment Center, which provides users with options for customizing stopping rules such as the minimum and maximum number of items to administer, and maximum standard error.

### Study limitations

This convenience sample was drawn from only 6 hospitals; it may not reflect the diversity of people with SCI living in the United States. Future studies should evaluate sensitivity to change, demonstrate known groups validity, and provide information on interpretability and meaningfulness.

### Clinical applications

Screening during outpatient visits allows clinicians to identify individuals with low levels of Ability to Participate or Satisfaction with Social Roles and Activities. Discussions with patients about the meaning and consequences of the scores could help guide referrals and treatment plans.

### Research opportunities

These 2 SCI-QOL social domain variables can be used in epidemiological studies to monitor population health and the effects of interventions designed to enhance participation, among other applications.

## Conclusions

The Ability to Participate and Satisfaction with Social Roles and Activities items provide state-of-the-art measures that can be administered using CAT or short forms. The measures are compatible with the PROMIS and Neuro-QOL family of measures. They complement the physical, emotional, and other social domain item banks.

### Suppliers

*Mplus Statistical Analysis with Latent Variables User's Guide* [computer program]. Version 6. Los Angeles: Muthen & Muthen; 2007.

*MULTILOG: Multiple, categorical item analysis and test scoring using item response theory* [computer program]*.* Chicago, IL: Scientific Software; 1991.

## Acknowledgements

We are indebted to David Victorson, Ph.D. for his assistance with conceptual and methodological input and Seung Choi, PhD for data analyses.

## Disclaimer statements

**Contributors** All authors have contributed significantly to the design, analysis and writing of this manuscript. The contents represent original work and have not been published elsewhere. No commercial party having a direct financial interest in the results of the research supporting this article has or will confer a benefit upon the authors or upon any organization with which the authors are associated.

**Funding** Funding was provided by the National Institutes of Health – Eunice Kennedy Shriver National Institute of Child Health & Human Development/National Center for Medical Rehabilitation Research and the National Institute of Neurological Disorders and Stroke (grant no. 5R01HD054659), and by the National Institute on Disability and Rehabilitation Research (grant nos. H133N110014, H133N110002, H133N110006, and H133N110020).

**Conflicts of interest** All SCI-QOL items and parameters are copyright © 2015 David Tulsky and the Kessler Foundation. All rights reserved. All Neuro-QOL items are copyright © 2008–2013 David Cella on behalf of the National Institute for Neurological Disorders and Stroke (NINDS). All items are freely available to the public via the Assessment Center platform (www.assessmentcenter.net). There are currently no plans for Dr. Tulsky or Kessler Foundation to profit from the use of the copyrighted material.

**Ethics approval** The Institutional Review Board at each site reviewed and approved this project.
